# HOW do we improve the testing of female ballistic body armour? – a comparison of roma plastilina no.1, 10% ballistic gelatine and sebs gel

**DOI:** 10.1007/s00414-025-03578-z

**Published:** 2025-09-23

**Authors:** Chris Malbon, Clare Knock, Debra J. Carr

**Affiliations:** 1 Centre for Defence Engineering, Cranfield University, Defence Academy of the United Kingdom, Shrivenham, Wiltshire SN6 8LA UK; 2 Formally 1, now at School of Applied and Health Science, London South Bank University, London, 103 Borough Road, SE1 0AA UK

**Keywords:** Back face signature (BFS), Behind armour blunt trauma, Breasts, Ballistic gelatine, SEBs

## Abstract

Body armour designed for use by police officers in England and Wales is currently tested using Roma Plastilina No1 (RP1) as the witness material for the measurement of back face signature (BFS). However, this material has limitations when testing body armour designed for females, as it is not possible to measure the BFS in the breast region due to the way the breast shapes are formed. Therefore, to enable measurement of BFS for females over the breast, an alternative backing material is required to form surrogate breasts and torso which would enable BFS to be measured. A comparison was conducted between RP1, 10% ballistic gelatine and a 30/70% styrene-etylene / butylene-styrene (SEBS) gel, using standardised ballistic test packs and two projectiles: DM11A1B2 9 mm FMJ at velocities 365 ± 10 ms^−1^; Remington R357M3 0.357” JSP at velocities 390 ± 10 ms^−1^. The results showed that there was a statistically significant difference identified in measured BFS among the three backing materials with both projectile types. RP1 had the overall smallest variance in measured BFS for both projectile types, however the limitation in being able to mould to create a breast shape is a major limiting factor. With 10% ballistic gelatine, when testing with the 0.357” projectile, a greater variance in measured BFS was shown compared to the other materials. The SEBS gel was consistent for the 0.357” projectile, but with the 9 mm projectile there was greater variance in results. Both 10% ballistic gelatine and SEBS gel would enable a moulded female test form to be created, however SEBS gel has a much longer shelf life and showed resistance to damage, although neither of these materials could be considered as a biofidelic substitute for breast tissue.

## Introduction

Body armour is commonly used by military and law enforcement personnel when there is a risk of a ballistic or stab attack. The level of resistance to threats provided by body armour can vary among users and roles [1]. Body armour can be designed to fit male or female torsos, more commonly referred to as unformed and formed armours, although not all body armour is available in both unformed and formed versions. Ballistic protective body armour is not only designed to resist perforation by projectiles, but also to reduce the risk of an injury or lethality from behind armour 3 blunt trauma (BABT), defined by Cannon as “…spectrum of non-penetrating injuries to the torso resulting from the impact of projectiles on personal armours.” [2].

Research into BABT has been extensively discussed in the open literature (e.g [3, 4]). However, very little of this research considered BABT over the female breast, or the potential injuries that may occur to the breast structure. Whilst these injuries in themselves may not be life threatening, they could be potentially life changing for a female user. The way in which the armour materials react to impact may also be affected by the dynamic response of breast tissue to an impact. This reaction of the armour materials is critical in the dissipation of energy from the impact and the prevention of the round penetrating through the body armour.

### Roma plastilina No1

**Fig. 1:**
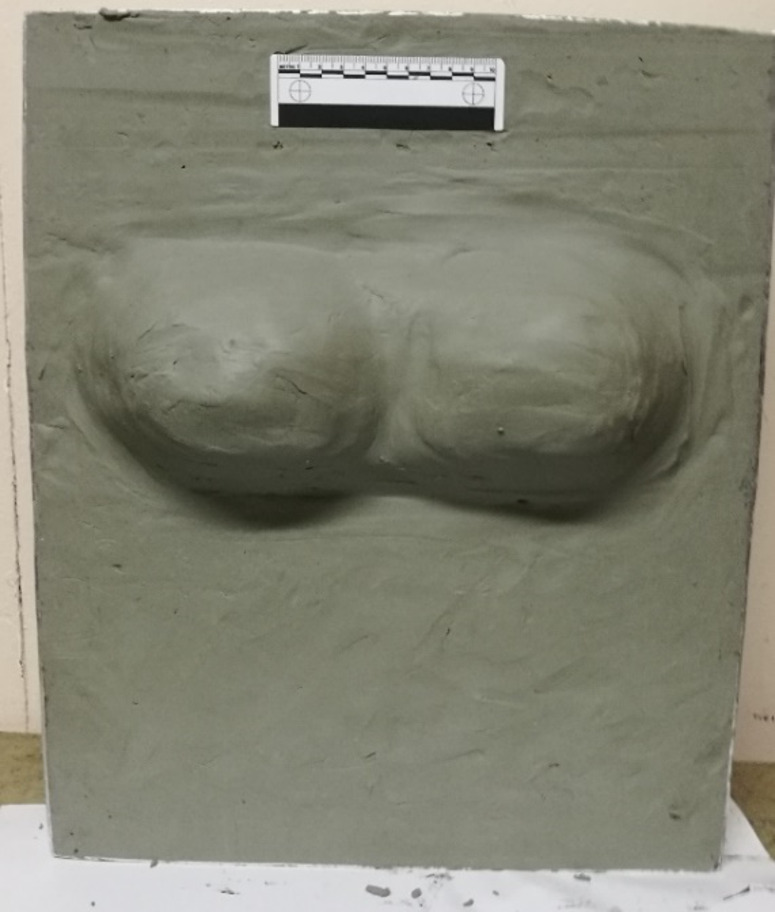
Example female formed shape made from RP1 on a flat backing tray (image produced by author)

**Fig. 2:**
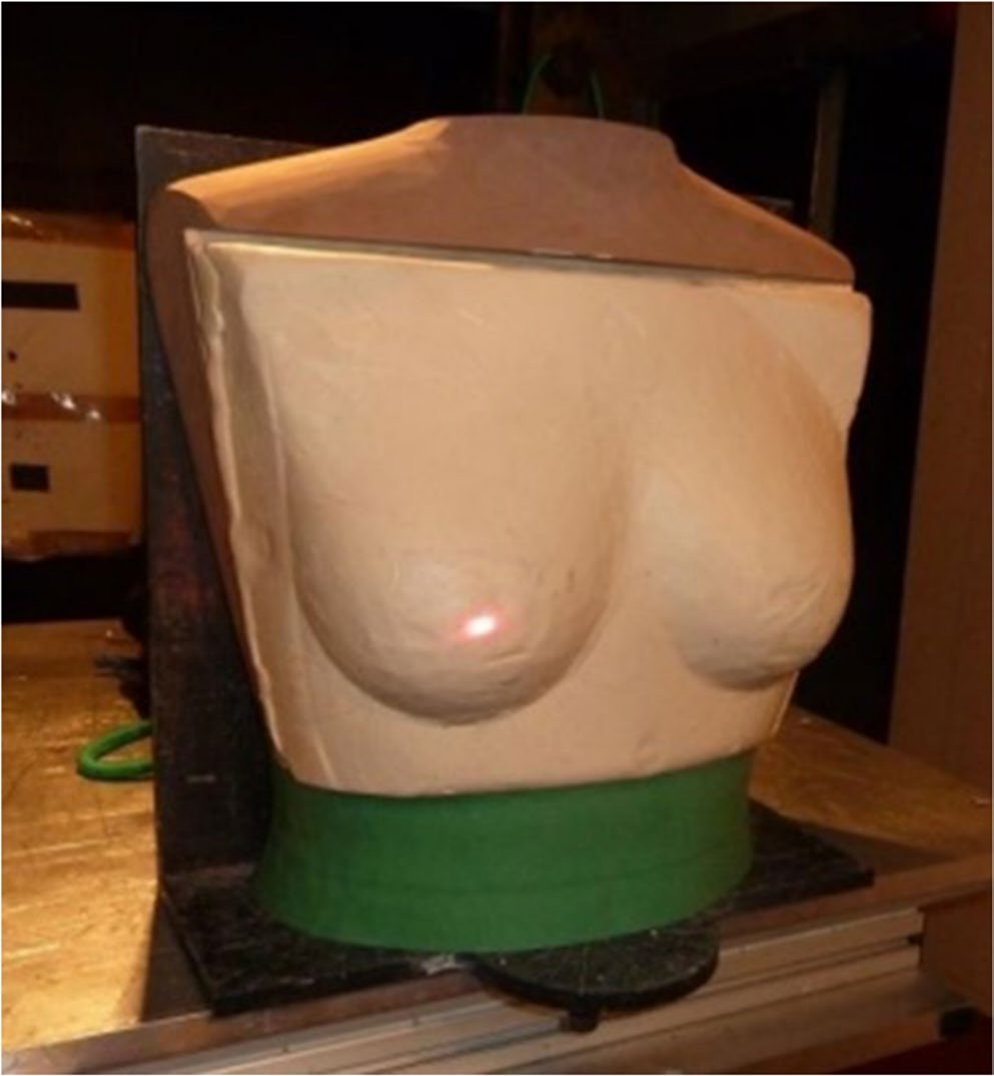
Example female test form as specified in home office body armour standard 2017 (image produced by author)

Since the 1970 s, the traditional material used when assessing the performance of body armour has been various forms of modelling clay, such as Roma Plastilina No1 (RP1) which is used in civilian standards in the United States of America and the United Kingdom [5, 6]. The clay provides a witness material that enables the measurement of any deformation of the body armour panel post testing, commonly referred to as back face signature (BFS). The current limits specified in the UK standard for the maximum depth of BFS for low velocity bullets, such as those from handguns, is 44 mm. For armour designed to protect against high velocity projectiles, such as rifle bullets, the limit is currently 25 mm. Unformed body armours, such as those typically used by males, are tested on flat blocks of modelling clay prepared in accordance with a relevant standard e.g [5–7]. However, formed body armours, such as those typically worn by females, have traditionally been tested on modified modelling clay blocks. Shapes made by free hand to fit the supplied test body armour approximating a bust, are stuck to the flat surface of the block used for testing unformed body armour, Fig. 1 [8]. More recently, in the UK the Home Office 2017 body armour standard introduced two female forms made from Plastiline^®^ 40^1^ [9]. Unlike RP1, Plastiline^®^ 40 can be melted a number of times to enable pouring into moulds, resulting in a standardised form leading to improve consistency in testing, Fig. 2 [5].

When testing unformed body armours the measurement of the resultant BFS in the modelling clay is feasible. However, when testing formed body armours this is not possible as the breast shapes created are unconstrained and collapse after impact. This results in only a pass or fail assessment based on whether or not the projectile perforates the body armour under test.

RP1 and Plastiline^®^ 40 are not biofidelic and the BFS recorded has no correlation to BABT [10]. The limits set in the standards for maximum BFS recorded in RP1 were based on torso shots onto body armour packs placed over ribs of goats [11, 12]. These shots were recreated on 20% ballistic gelatine, which at the time was commonly used in the United States of America for wound ballistic research, and on several clay-based materials. The selection of RP1 was based on it having a similar performance to 20% ballistic gelatine and being low cost, convenient, and not requiring the use of expensive high-speed filming. When RP1 was selected, formed body armour was not tested and breast tissue was not considered. Also, RP1 is manufactured as a modelling clay for use by artists, rather than a consistent backing material for testing of body armour. The formulation of RP1 is not tightly controlled and its properties have varied since 1975 changing its performance as a backing material for testing body armour [13]. To manage this variance in material properties, calibration methods were defined in international body armour standards. By the use of temperature preconditioning, the response of RP1 can be controlled to meet the requirements of a standard.

Very little work has been published concerning backing materials used for assessing ballistic impacts on body armour for female users. Research on underwired bras and potential injury from a non-perforating ballistic impact on a body armour was considered using female torsos made from PermaGel™ and from RP1 [14]. The research noted small BFS indentations in the RP1, however only two shots were reported. Overall, the research did not identify an obvious risk of additional injury due to an underwired bra after impact by the test projectile, however, they did not consider BFS in the PermaGel™. PermaGel™ is reportedly not a good tissue simulant for wound ballistics studies compared to ballistic gelatine [15]. The depth of penetration of a 5.5 mm ball bearing into PermaGel™ was shorter at slower velocities (v = 250 ms^−1^, mean difference of −32.77 mm) and greater at faster velocities (v = 500 ms^−1^, mean difference of 33.5 mm) compared to 10% ballistic gelatine.

Work by Forster et al. investigated bust surrogates for use in stab testing of body armour [16]. A stack of neoprene circles of decreasing sizes was used to create bust shapes, but limited testing was reported. The concept was simple to implement, however human breasts are not perfect circles. The foam circles were placed on a flat backing surface which would not enable the body armour to form to a natural shape. The limited stab testing conducted resulted in one failure when testing on the foam breast surrogate compared to no failures on the RP1 breast surrogate; between the busts two out of three failures were reported for the foam. Careful positioning of the body armour was required to ensure best fit on both surrogates. Whilst the approach was simple, testing of a body armour on a non-natural shape may affect the results.

### SEBS gel

SEBS gel was first discussed in the open literature in 2010 as an alternative to ballistic gelatine in the assessment of dynamic BFS [17]. SEBS gels are made from commercial thermoplastic elastomers consisting of triblock co polymers; styrene-ethylene/butylene-styrene which forms a transparent block when mixed with mineral oil [18]. A comparison of 30/70% by mass SEBS gel to 20% ballistic gelatine showed comparative results of back face signature for impacts on body armour between 600 ms^−1^ to 800 ms^−1^ [19]. The main advantage of the SEBS gel over ballistic gelatine was its reusability and stability over extended time periods, with over 100 shots fired at the same SEBS gel block over a five-day period with no apparent change in results, compared to a very limited usable life span for ballistic gelatine of a couple of days.

More recent work has compared impacts on SEBS gel to human response to injury, particularly to less lethal kinetic impacts [18–21]. However, there is not yet sufficient data to determine if SEBS gel can be used for assessment of BABT for formed body armours.

### 10% ballistic gelatine

Gelatine has been used to represent human tissue in wound ballistics studies; it is commonly used in two different formulations, 10% and 20% (mass of gelatine powder to water).

Gelatine is typically produced by the extraction of collagen from tissue and its use as a tissue surrogate for wounds ballistics research has been well reported in the open literature. A comprehensive overview of the use of ballistic gelatine in wound ballistics is available [22]. Work by Mabbott et al. compared 10% and 20% gelatine to porcine thorax for wound ballistics [23]. 10% ballistic gelatine provided a more representative depth of penetration (DoP) than 20% ballistic gelatine when considering faster velocity projectiles (5.56 × 45 62 grain; 10%, V_mean_ = 847 ms^−1^ DoP_mean_ = 426 mm; 20%, V_mean_ = 845 ms^−1^, DoP_mean_ = 296 mm; thorax model, V_mean_ = 846 ms^−1^, DoP_mean_ = 460 mm).

The performance of gelatine in wound ballistic studies is affected by temperature and aging (e.g [24, 25]). The temperature of gelatine blocks affects the mechanical properties including penetration resistance and needs to be carefully controlled.

The use of ballistic gelatine for assessment of BABT was first discussed by Metker et al. who used 20% gelatine to develop a method for measuring BFS behind body armour [26]. The selection of gelatine was to enable the visualisation and measurement of BFS using a high-speed camera, although no details of the exact gelatine used or why 20% gelatine was selected were included in the report. Work published in 1977 compared the BFS behind body armour in 20% gelatine to the injuries seen in goats’ thorax [12]. The 20% gelatine blocks had a larger BFS than that for goat thorax; interestingly in the data presented, RP1 had a very similar response to the gelatine blocks.

### Aim

The aim of this work was to compare potential materials for the testing of female body armour with respect to ballistic resistance of body armour and BABT.

In this paper the commonly used testing material RP1 was compared against:

- 30%/70% SEBS gel.
- 10% (by mass) gelatine. This concentration was selected based on the work by Mabbott et al. (2013) [15].

## Method

A standardised test protocol was developed to establish a data set to enable a comparison of the three materials to be conducted.

### Test panels

**Fig. 3:**
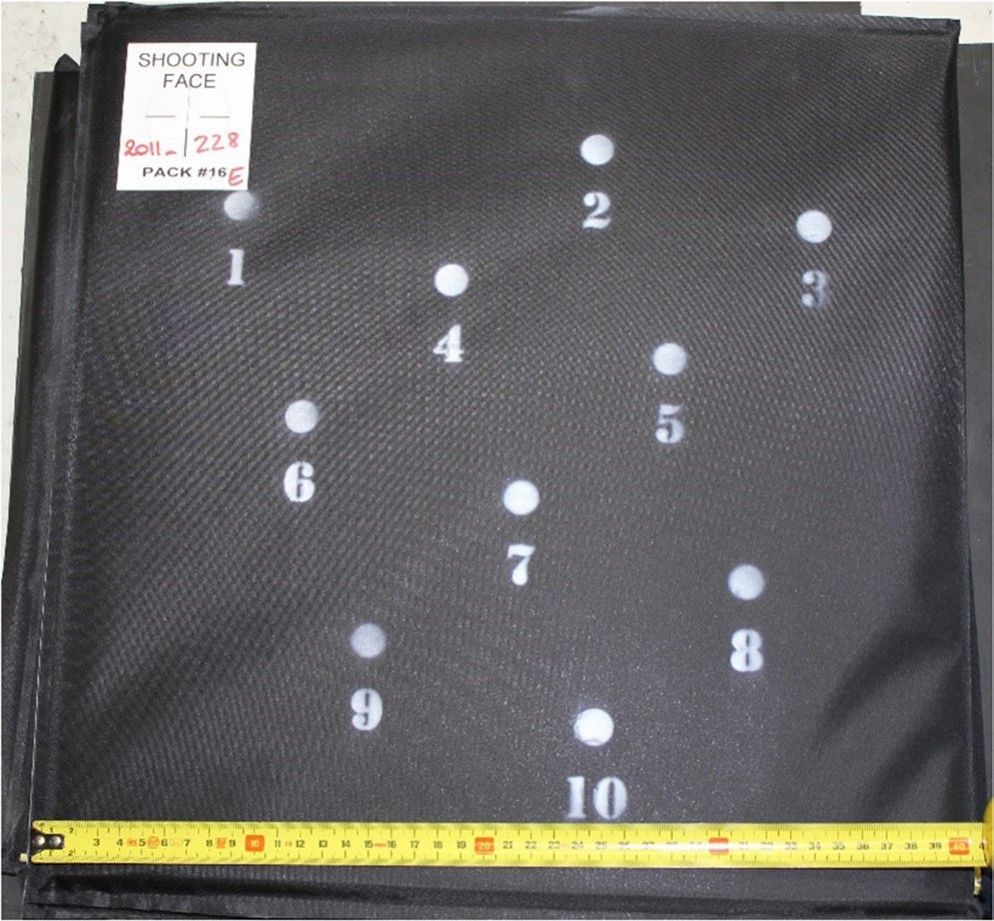
Shot location marked on test panel

All ballistic testing was conducted using a single batch of test panels manufactured by DuPont^®^, specified to have a mean BFS on RP1 of 25 mm, and each measuring 400 × 400 mm. The panels consisted of 10 layers of Kevlar^®^ XP102 with a single layer of 3 mm foam on the non-strike face. Each test panel was marked with the same ten shot pattern (Fig. 3). Each shot location was a minimum of 50 mm from the edge of the panel and at least 100 mm from any other shot location, all shot locations were on a different horizontal and vertical axis. A panel was placed on one of the three test simulants and subjected to ballistic testing using the projectiles described below.

### Projectiles

Testing was conducted using the test projectiles, velocities, and range conditions (temperature and humidity) as specified in the HOSDB Body Armour Standard 2007 Part 2, at the HG1a protection level (Table 1; [8]).

**Table 1:** Test round details and velocities [8]

Calibre	Description	Projectile mass	Range (m)	Velocity (ms^−1^)
9 mm	9 mm Full Metal Jacket (FMJ) Dynamite Nobel DM11A1B2	8.0 g (124 grain)	5	365 ± 10
0.357”	0.357” Magnum Jacketed Soft Point (JSP) Remmington R357M3	10.2 g (158 grain)	5	390 ± 10

### Backing material – Roma plastilina No1

Two steel trays (420 mm x 350 mm x 100 mm) were filled and prepared from a single batch of new RP1 material and conditioned in accordance with Home Office Scientific Development Branch (HOSDB) body armour standard 2007 [8]. The trays were rotated throughout testing, each being refilled and smoothed with preconditioned material from the same initial batch of material before being placed back into the conditioning chamber as described in the test standard. Each tray was tested for compliance with calibration checks before each batch of testing (Table 2). If a tray was outside calibration, it was refilled, smoothed and place back in the conditioning chamber for a minimum of 30 min before being rechecked.

**Table 2:** Calibration check of RP1 tray

Test object	Drop height (m)	Spacing	Requirement
Steel sphere Diameter 63.5 ± 0.05 mm Mass 1.043 ± 0.005 kg	1.5 ± 0.02 m	75 mm from the edge of the tray to any indent centre 100 mm between indent centres	Mean depth of indent 15 ± 1.5 mm

During testing, no test shot was conducted closer than 50 mm to the edge of the tray or 100 mm from a previous impact point. The depth of the indent in the RP1 was measured at its deepest point using a calibrated digital calliper to determine the BFS to provide the data for analysis as described in Sect. 3.7.

### Backing material – 10% ballistic gelatine

Type 3 (265 bloom) ballistic photographic grade gelatine from the same batch was used to make ten blocks of 10% ballistic gelatine. Blocks were manufactured in accordance with the instructions in Mabbott’s PhD thesis [27, p262].

Once set, each block was cut in half lengthways to create blocks of size 250 mm x 300 mm x 250 mm. Calibration of the blocks was performed on removal from cold storage using 4.5 mm steel ball bearings (average mass of 0.372 g) fired at velocities between 120 ms^−1^ and 190 ms^−1^ and depth of penetration (DoP) measured. This was compared in the spirit off the data from Jusilla’s work for comparison and between test blocks for consistency [25]. If the DoP was outside the range detailed in the work by Jussila, the block was excluded from the study. The temperature of the blocks and the firing range was not recorded.

During testing no two impacts occurred on the same location and the block was rotated to an alternative side if any damage was noted.

Each shot was recorded using a Phantom high-speed camera at 21,000 frames per second, mounted perpendicular to the side of the block. Post-test analysis of the recordings was conducted using Phantom CineView (CV) software^2^ to provide the data for analysis as described in Sect. 3.7. Calibration was achieved through a photographic scale and set for each high-speed video.

### Backing material – SEBS gel

A single SEBS gel block of size 250 mm cubed, composition 30% mass polymer (Kraton G1652) and 70% mass mineral oil (Primol 352) was used. The block was manufactured and consistency checked in accordance with the process detailed by Bracq et al. [18]. The SEBS gel did not require preconditioning before use and all test shots were conducted on the same location.

Each shot was recorded using a high-speed camera at 10,000 frames per second, mounted perpendicular to the side of the block with a photographic scale visible. The captured video was processed using an automated algorithm developed by Centre de Recherche et d’Expertise de la Logistique (CREL) to provide the data for analysis as described in Sect. 3.7.

### Test method

Testing was conducted as described in the HOSDB body armour standard 2007 for ballistic testing [8] at three separate test facilities. RP1 testing was conducted by the Home Office Centre for Applied Science and Technology (CAST), St Albans (UK), 10% ballistic gelatine was tested at the Impact and Armour Group at Cranfield University, Shrivenham (UK) and SEBS gel was tested by CREL in Paris (France). Each test facility was provided with ten pre marked test panels for use during the trial.

### Data analysis

The data was checked to ensure that shot velocities were in accordance with of the values specified in Table 1, those outside of specification were not included in further analysis. The number of usable test shots per projectile and backing material type is shown in Table 3.

**Table 3:** Number of samples available post validation checks

Material	Projectile type	Number of test packs used	Total Number of valid shots
Roma Plastilina No 1	9mm	5	30
	0.357"	5	43
10% Ballistic gelatine	9mm	5	46
	0.357"	5	47
SEBS Gel	9mm	4	29
	0.357"	5	33

For 10% ballistic gelatine and SEBS gel, the depth and diameter of the BFS were collected at each frame of video until the maximum depth was identified. From this data the rate of change (acceleration) for depth was calculated using Equation 1. An estimate of volume of BFS was calculated based on the assumption the shape could be considered a spherical cap^3^, Equation 2.

1 $$\begin{array}{cc}a=\frac{\triangle\nu}{\triangle t}&a:acceleration\\&\nu:\frac{\nu d}{\triangle t}(velocity)\\&t:time\\&d:dis\tan ce\end{array}\\\\\\\\\\\\\\\\$$

2


The following rule was applied when reviewing dynamic BFS data for 10% ballistic gelatine and SEBS gel.

- Test packs which had BFS outliers identified through residual analysis were excluded.

The following analysis of dynamic material behaviour was conducted.

- Within material comparison of dynamic BFS depth and time and velocity to peak BFS (Sect. 4.3).
- Between projectile type comparison of dynamic BFS surface diameter (Sect. 4.4).
- Between projectile type comparison of dynamic BFS volume (Sect. 4.5).
- Rate of change in BFS depth (Sect. 4.6).

### Statistical tests

All statistical analysis was conducted using SPSS^4^. Only statistically significant results are discussed in the results section. A two-way analysis of variance was conducted to explore the impact of backing material and shot location of maximum BFS for both projectile types. Statistical test for homogeneity of variance was conducted. Where a statistically significant variance was identified (*p* < 0.05) a post-hoc comparison was conducted using Tukey Honestly Significant Difference test (Tukey HSD).

## Results^5^

For all backing materials the data was analysed for variance within and among test packs as well as amongst backing materials. For both SEBS gel and 10% ballistic gelatine, further analysis was conducted looking at the dynamic response of the materials to impact captured using high speed video. The dynamic response analysis was not possible for the RP1 due to the nature of the material not being transparent preventing high speed video recording.

### Recorded BFS

Descriptive statistics for recorded BFS for both projectiles for shots within velocity tolerance were created, Table 4.

**Table 4:** Descriptive statistics for BFS

Projectile type	Descriptive	RP1	10% ballistic gelatine	SEBS gel
**9 mm**	Mean BFS (mm)	22.8	30.8	36.2
	Standard deviation	3.1	4.6	4.6
	Max BFS (mm)	30.9	42.3	44.7
	Min BFS (mm)	17.6	21.5	29.5
	N	30	46	29
**0.357**”	Mean BFS (mm)	25.2	44.4	39.9
	Standard deviation	3.7	8.4	5.3
	Max BFS (mm)	32.5	67.3	52.8
	Min BFS (mm)	18.9	31.5	33.4
	N	43	47	33

For the 9 mm projectile, the differences in mean data for all three materials was minimal (mean BFS RP1 = 22.8 mm, 10% ballistic gelatine = 30.8 mm, SEBS gel = 36.2 mm) when compared to the 0.357” projectile (mean BFS RP1 = 25.2 mm, 10% ballistic gelatine = 44.4 mm, SEBS = 39.9 mm). 10% ballistic gelatine had the greatest spread of results, especially at for the 0.357” projectile (BFS 31.5 to 67.3 mm). The spread of results for RP1 was the smallest for both projectiles (9 mm, BFS = 17.6 to 30.9 mm; 0.357”, BFS = 18.9 to 32.5 mm).

### Effect of backing material and shot location on maximum BFS depth for both 9 mm and 0.357” projectiles

Homogeneity of variances of maximum BFS for backing material type and shot location on test panel was tested using Levene’s test of equality of error, which identified that there were significant results (*p* < 0.01) for both projectile types. A more stringent significance level of 0.01 was therefore applied to the output of the two-way ANOVA.

There was no statistically significant interaction effect among backing material and shot location on maximum back face signature, (9 mm, F_(18, 66)_ = 2.155, p = NS, 0.357”, F_(18, 93)_ = 1.186, p = NS). There were statistically significant main effects for both backing material, (9 mm, F_(2, 66)_ = 142.123, p < 0.001, 0.357”, F_(2, 93)_ = 171.766, p < 0.001) and for shot location, (9 mm, F_(9, 66)_ = 7.526, p < 0.001, 0.357”, F_(9, 93)_ = 8.691, p < 0.001). For both projectiles, the effect size for the backing material, (9 mm, partial eta squared = 0.812, 0.357”, partial eta squared = 0.787), was greater than for shot location, (9 mm, partial eta squared = 0.506, 0.357”, partial eta squared = 0.457).

Post-hoc comparisons conducted using Tukey HSD for the effect of backing material type, within projectile, on maximum BFS for RP1 (9 mm, M = 22.7 mm, SD = 3.1 mm, 0.357”, M = 25.2 mm, SD = 3.7 mm), gelatine (M = 29.3 mm, SD = 3.6 mm, 0.357”, M = 44.4 mm, SD = 8.5 mm), and SEBS (M = 36.2 mm, SD = 4.6 mm, 0.357”, M = 39.9 mm, SD = 5.4 mm) were all statistically significantly different from each other.

For shot location, Tukey HSD post-hoc comparison indicated that the maximum BFS for all shot locations, within projectile type, apart from 2 to 8 had a significant difference to at least two other shot locations, Tables 5 and 6.

**Table 5:** Comparison of effect of shot location on BFS for 9 mm, all backing materials

Shot location (mean and SD; mm)	Significant difference to (mean and SD; mm)
1 (M = 31.2, SD = 5.4)	4 (M = 26.3, SD = 5.6)
	7 (M = 26.6, SD = 6.3)
3 (M = 34.7, SD = 6.8)	4 (M = 26.3, SD = 5.6)
	5 (M = 28.3, SD = 7.4)
	6 (M = 27.6, SD = 4.5)
	7 (M = 26.6, SD = 6.3)
	9 (M = 28.0, SD = 6.6)
4 (M = 26.3, SD = 5.6)	1 (M = 31.2, SD = 5.4)
	3 (M = 34.7, SD = 6.8)
	10 (M = 34.1, SD = 7.1)
5 (M = 28.3, SD = 7.4)	3 (M = 34.7, SD = 6.8)
	10 (M = 34.1, SD = 7.1)
6 (M = 27.6, SD = 4.5)	3 (M = 34.7, SD = 6.8)
	10 (M = 34.1, SD = 7.1)
7 (M = 26.6, SD = 6.3)	1 (M = 31.2, SD = 5.4)
	3 (M = 34.7, SD = 6.8)
	10 (M = 34.1, SD = 7.1)
9 (M = 28.0, SD = 6.6)	3 (M = 34.7, SD = 6.8)
	10 (M = 34.1, SD = 7.1)
10 (M = 34.1, SD = 7.1)	4 (M = 26.3, SD = 5.6)
	5 (M = 28.3, SD = 7.4)
	6 (M = 27.6, SD = 4.5)
	7 (M = 26.6, SD = 6.3)
	9 (M = 28.0, SD = 6.6)

**Table 6:** Comparison of effect of shot location on BFS for 0.357”, all backing materials

Shot location (mean and SD; mm)	Significant difference to (mean and SD; mm)
1 (M = 42.3, SD = 11.5)	4 (M = 33.6, SD = 10.6)
	5 (M = 30.7, SD = 7.1)
	6 (M = 32.6, SD = 7.9)
	7 (M = 32.8, SD = 9.6)
3 (M = 43.9, SD = 12.2)	4 (M = 33.6, SD = 10.6)
	5 (M = 30.7, SD = 7.1)
	6 (M = 32.6, SD = 7.9)
	7 (M = 32.8, SD = 9.6)
	9 (M = 34.7, SD = 9.5)
4 (M = 33.6, SD = 10.6)	1 (M = 42.3, SD = 11.5)
	3 (M = 43.9, SD = 12.2)
5 (M = 30.7, SD = 7.1)	1 (M = 42.3, SD = 11.5)
	3 (M = 43.9, SD = 12.2)
	10 (M = 41.5, SD = 14.5)
6 (M = 32.6, SD = 7.9)	1 (M = 42.3, SD = 11.5)
	3 (M = 43.9, SD = 12.2)
	10 (M = 41.5, SD = 14.5)
7 (M = 32.8, SD = 9.6)	1 (M = 42.3, SD = 11.5)
	3 (M = 43.9, SD = 12.2)
	10 (M = 41.5, SD = 14.5)
9 (M = 34.7, SD = 9.5)	3 (M = 43.9, SD = 12.2)
10 (M = 34.1, SD = 7.1)	5 (M = 30.7, SD = 7.1)
	6 (M = 32.6, SD = 7.9)
	7 (M = 32.8, SD = 9.6)

The initial analysis of BFS data showed the following.

- Within backing material, for both projectiles, there was no statistically significant difference in result between shot locations.
- Amongst backing materials there was a statistically significant difference in results.
- Amongst shot locations, for both projectiles across all backing materials, there was a significant difference in results apart from shot locations 2 and 8.

The choice of backing material had the largest effect on the difference in BFS amongst all the variables tested (backing material, shot location, projectile type).

### Within material comparison of dynamic BFS depth and response rate

**Fig. 4:**
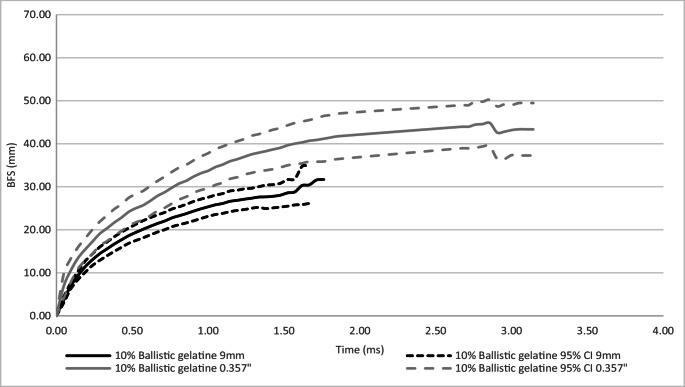
Comparison of 9 mm and 0.357” mean dynamic depth BFS − 10% ballistic gelatine

**Fig. 5:**
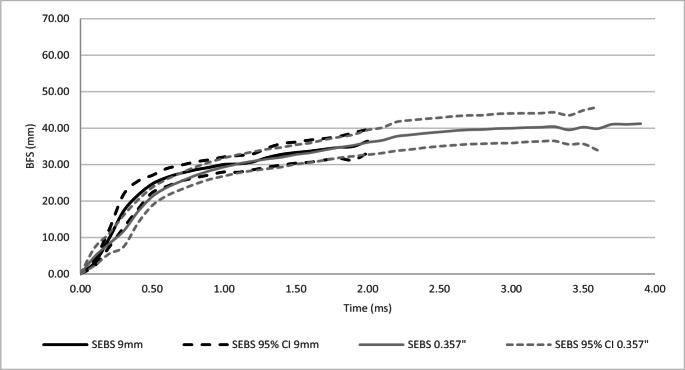
Comparison of 9 mm and 0.357” mean dynamic depth BFS – SEBS gel

A comparison of dynamic mean peak BFS and response curve for 10% ballistic gelatine and SEBS gel as a result of impacts from 9 mm and 0.357” projectiles was conducted, Figs. 4 and 5 respectfully and Table 7.

**Table 7:** Maximum depth BFS data for SEBS gel and 10% ballistic gelatine

Material	Projectile (KE) (*n*)	Peak BFS (mm) (95% CI)	Time to peak BFS (ms)
10% ballistic gelatine	9 mm (533 J) (*n* = 10)	35 (± 4.3)	1.86
	0.357” (776J) (*n* = 10)	49 (± 4.8)	3.38
SEBS gel	9 mm (533 J) (*n* = 5)	39 (± 1.54)	2.4
	0.357” (776 J) (n = 10)	41 (± 5.9)	3.9

The 10% ballistic gelatine for both the 9 mm and 0.357″ projectiles reached peak BFS faster (1.86 ms, 3.38 ms) when compared to SEBS gel (2.4 ms, 3.9 ms). However, Fig. 4 shows that 10% ballistic gelatine had a different response rate for the two projectiles, whereas Fig. 5 shows that SEBS gel had a very similar response to both projectiles. Due to its higher kinetic energy, a faster response rate was expected for the 0.357” as occurred in the 10% ballistic gel. The lack of noticeable difference in the SEBS gel indicates it was stiffer than the 10% ballistic gelatine, supporting the initial analysis on the distribution of results, Sect. 4.1.

For the 9 mm projectile, 10% ballistic gelatine responded quicker for the first 0.2 ms than the SEBS gel, however, at that point the reaction of the 10% ballistic gelatine slowed compared to SEBS gel. For the 0.357” projectile, the 10% ballistic gelatine had a more rapid response throughout the duration of the response compared to SEBS gel.

### Between projectile type comparison of dynamic BFS mean surface diameter

**Fig. 6:**
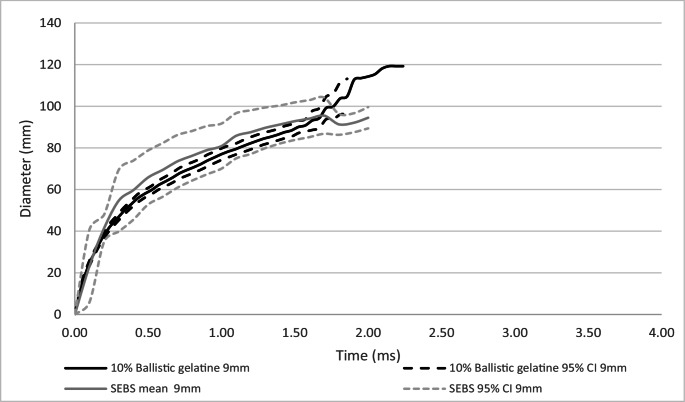
Comparison of dynamic BFS mean diameter with 95% confidence intervals (95% CI) on SEBS gel and 10% ballistic gelatine– 9 mm projectile

**Fig. 7:**
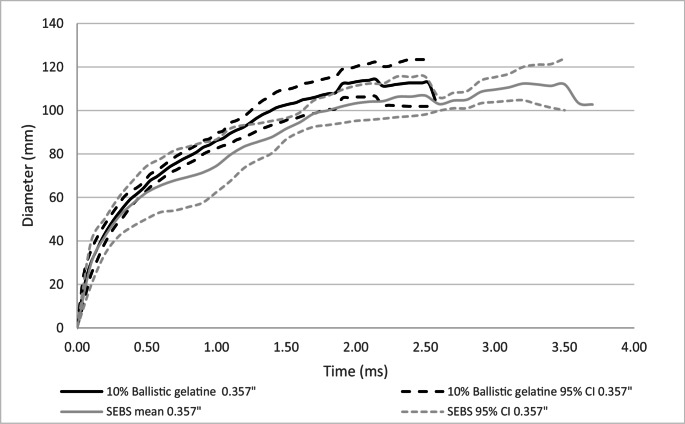
Comparison of dynamic BFS mean diameter with 95% confidence intervals (95% CI) on SEBS gel and 10% ballistic gelatine – 0.357” projectile

A comparison of dynamic BFS mean diameter at the surface of the backing material for 10% ballistic gelatine and SEBS gel within projectile type was conducted, Figs. 6 and 7.

For the 9 mm projectile, both 10% ballistic gelatine and SEBS gel had very similar mean diameters throughout the impact duration, Fig. 6. However, the maximum mean diameter for ballistic gelatine was greater than for the SEBS gel (mean 119 mm, 94 mm respectively). For the 0.357” projectile, the maximum mean diameters for both 10% ballistic gelatine and SEBS gel were very similar (113 mm, 112 mm respectively). However, as the duration of the impact for the SEBS gel was longer than for the 10% ballistic gelatine, the increase in diameter over time was slower than for the 9 mm projectile.

For both BFS depth and diameter, the 0.357” projectile had higher mean peak values in both the 10% ballistic gelatine and the SEBS gel compared to the 9 mm projectile. The time to peak BFS depth was longer for SEBS gel for both projectile types compared to the 10% ballistic gelatine, which generally also showed a slower mean response rate in the curves.

A further investigation was conducted to understand if there was a relationship between the maximum depth and diameter of BFS. A Pearson product-moment correlation coefficient test was conducted to assess the relationship and if it was significant, Table 8.

**Table 8:** Pearsons product-moment correlation coefficient test - depth vs. diameter

Backing material	Projectile	Pearsons product-moment correlation
10% Ballistic gelatine	9 mm	*r* = 0.925, *n* = 9, *p* < 0.001
	0.357”	*r* = 0.513, *n* = 10, *p* > 0.05
SEBS	9 mm	*r* = 0.522, *n* = 10, *p* > 0.05
	0.357”	*r* = −0.497, *n* = 9, *p* > 0.05

There was a statistically significant, strong positive correlation between depth of BFS and diameter for the 9 mm projectile on the 10% ballistic gelatine. However, for the 0.357” projectile on 10% ballistic gelatine, and both projectiles on SEBS, no statistically significant correlation was identified.

### Between projectile type comparison of dynamic BFS mean volume

The mean volume of the BFS signature was estimated from the data obtained for both depth and diameter, using the assumption that the shape of the witnessed BFS is a spherical cap. This enabled a comparison of the mean volume to be conducted using Eq. 2.

**Fig. 8:**
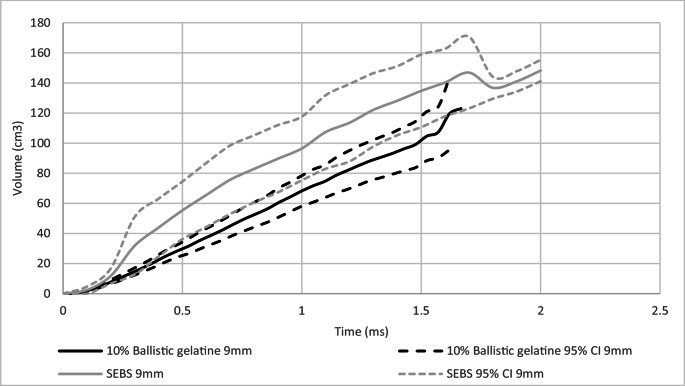
Comparison between SEBS gel and 10% ballistic gelatine of dynamic BFS mean estimated volume of BFS with 95% confidence intervals (95% CI) for 9 mm

**Fig. 9:**
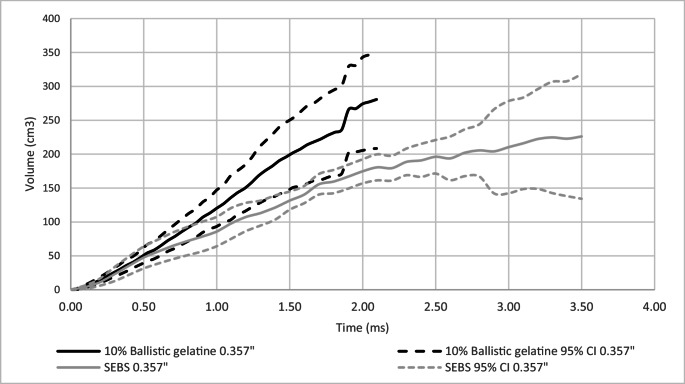
Comparison between SEBS gel and 10% ballistic gelatine of dynamic BFS mean estimated volume of BFS with 95% confidence intervals (95% CI) for 0.357”

A comparison of dynamic BFS mean volume, within projectile type on 10% ballistic gelatine and SEBS gel was conducted, Figs. 8 and 9.

The volume of the maximum mean BFS for the 9 mm projectile was greater for SEBS gel compared to 10% ballistic gelatine, Fig. 8 (148 cm^3^, 120 cm^3^ respectively). For the 0.357” projectile the volume of the maximum mean BFS was greater for the 10% ballistic gelatine than the SEBs gel, Fig. 9 (281 cm^3^, 226 cm^3^).

### Rate of change in BFS mean depth

**Fig. 10:**
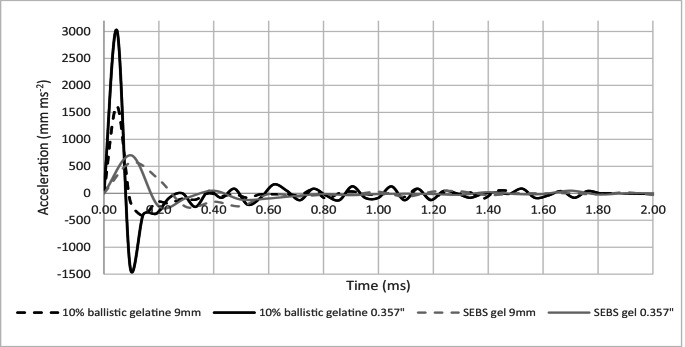
Acceleration of BFS mean depth in 10% ballistic gelatine and SEBS gel in response to impacts on armour panels from 9 mm and 0.357” projectiles

To investigate further the difference in response of the two materials, the rate of change (acceleration) of the BFS depth for each projectile type was calculated using Eq. 1 and is shown in Fig. 10.

The 10% ballistic gelatine had a far greater initial response to impact for both the 9 mm and 0.357” projectile when compared to SEBS gel. In both materials for the 9 mm projectile, the initial response lasted approximately 0.3 ms before reducing to a ‘steady’ state. For the 0.357” projectile on SEBS gel the response also settled to a ‘steady’ state around 0.3 ms, but for 10% ballistic gelatine, it showed evidence of ‘oscillations’ in its response to over 1 ms.

## Discussion

The purpose of this study was to identify variances among three different backing materials, RP1, 10% ballistic gelatine, and SEBS, for assessment of BFS, and the consideration for their use in the testing of formed body armour designed for female wearers. Each backing material was tested using two projectiles, DM11A1B2 9 mm FMJ at velocities 365 ± 10 ms^−1^; Remington R357M3 0.357” JSP at velocities 390 ± 10 ms^−1^.

The key findings from this study were.

- There was statistically significant variance among RP1, 10% ballistic gelatine and SEBS gel in the measurement of mean BFS for both projectiles considered.
- For all three backing materials, 10% ballistic gelatine exhibited the greatest variation in BFS.
- RP1 had the smallest distribution of results of the three materials.
- The mean BFS was greater for SEBS gel at lower impact energies and greater for 10% ballistic gelatine at higher energies.
- The mean diameter of the BFS was similar in SEBS gel and 10% ballistic gelatine.
- The mean volume of the BFS signature followed the same pattern as for the BFS.
- 10% ballistic gelatine was more energy sensitive and responsive to change than SEBS gel.

The data presented in this paper is based on a total of 228 test shots (9 mm, n = 105, 0.357” n = 123) which has enabled a comprehensive comparison to be conducted.

The statistically significant variance in results among the BFS measured in the three backing materials means it is not possible to simply swap one material for another when it comes to the assessment of BFS.

This research only considered slower velocity rounds typically used in handguns. At faster velocities and hence higher energies, it is unknown how 10% ballistic gelatine would perform in measuring BFS. The data presented in Fig. 4 shows that when testing with the 0.357” projectile there was an increase in the variance of the measured BFS compared to the 9 mm projectile. The higher energy of the 0.357” projectile at impact compared to the 9 mm projectile (776 J and 533 J respectively) may account for this. This effect could be exaggerated if testing higher energy projectiles (e.g. 7.62 × 39 mm or 5.56 × 45 mm).

The results for RP1 had the smallest spread of BFS depths compared to the other materials for both the 9 mm and 0.357” projectiles. On closer examination of the results, it was suspected that this was due to the cooling of the RP1 during testing. Work by Seppala et al. has demonstrated that as RP1 cools it becomes stiffer, which relates to a smaller indent being formed from the same impact energy [13]. This demonstrates that when working with RP1 time is critical to the completion of testing while the material is still showing similar levels of strain rate sensitivity.

The mean diameter of the BFS in both 10% ballistic gelatine and SEBS gel within each projectile type were closely matched, Figs. 6 and 7. Considering this in relation to testing for formed body armour, particularly for female breast forms, the diameter of the BFS may be significant. A large diameter BFS may be greater than the diameter of the test form, which could potentially affect the resultant depth of BFS measured when used for testing of impacts over the breast.

Whilst the diameter of the BFS was relatively consistent between SEBS gel and 10% ballistic gelatine, the depth of the BFS varied, which when calculating the estimated volume produced distinct differences between the materials. If energy transfer as a potential mechanism for assessing BABT related injury potential was to be considered as a method, an understanding of effect of the variance in volume has occurred would be needed.

RP1 is not ideal for the testing of formed body armours designed for the female form, due to the difficulty in shaping anatomically correct breast shapes and its lack of biofidelity especially when compared to human breast tissue.

When made, ballistic gelatine is in a liquid form before it is allowed to set and can therefore be used in various shaped moulds, therefore making a suitable female form with breast surrogates practicable. Whilst the potential of using 10% ballistic gelatine may be suitable for low velocity 9 mm projectiles, it has been shown that as the energy of the projectile increases the measurement of BFS in 10% ballistic gelatine becomes more variable. Additionally, ballistic gelatine has only a short workable life (typically 2 to 3 days), before it is not within calibration and must be replaced, resulting in the constant production of new blocks. Also, in a similar way to RP1, the properties of ballistic gelatine are sensitive to temperature, meaning the workable time after removal from a conditioning chamber limit its effectiveness in a test facility. This makes the use of ballistic gelatine a labour-intensive and expensive option.

SEBS gel may be a viable alternative to both RP1 and 10% ballistic gelatine, as it is stable at room temperature, has an extended life and has a range of methods to ensure between batch consistency. In the same way as gelatine, it is also mouldable, so could be used as a potential surrogate for the female form. However, with the formulation used in this work (30/70), at the lower energy of the 9 mm projectile, it had the greatest spread of results in measured BFS which is not ideal for testing body armour. The relationship between diameter and depth of BFS would also need to be investigated to understand how the material is responding, and if that affects the potential to use energy transfer to assess potential BABT injuries.

## Conclusions

This work has presented a comparative data set on three different materials: RP1; 10% ballistic gelatine; SEBS gel, which have been used for the assessment of BFS. The results have been considered with reference to the female form. This work has highlighted that whilst RP1 is the established medium for most current test standards, there is variation in the measured BFS among test shots on the same armour panel. This variation in measured BFS (9 mm min = 17.6 mm, max = 30.9 mm; 0.357” min = 18.9 mm, max = 32.5 mm) could be enough to wrongly pass or fail a body armour^6^. RP1 does provide a low cost, simple method for the testing of body armour that can be conducted without the need for specialist equipment such as high-speed cameras. Both 10% ballistic gelatine and SEBS gel are not currently used with any published standard, so it is not possible to comment on how the result may affect any certification testing. Both 10% ballistic gelatine and SEBS gel would require the use of high-speed camera and post-test analysis of the video footage. Ballistic gelatine also has the disadvantage of having a limited shelf life once made, which potential makes it an expensive option in terms of labour to utilise.

At this current time, no one material is the optimal for testing body armour. However, the possibility of being able to have a material that has a more human like response to impact and can be formed to create breast surrogates in a lifelike shape has many advantages. More research is urgently required to understand the potential injury mechanisms of the breasts, and the effect breast tissue has on the properties of armour materials.

Of the materials tested in this research, SEBS gel is the most usable for further development of a test form that enable more realistic testing of body armour, particularly those designed for female wearers, with its ability to be moulded and its long usable life span.

## Footnotes

1 G.Lalo, Herbin Sueur SA, France.

2 Version 3.5.792.0.

3 “A portion of a ball cut from it by a horizontal plane is called a spherical cap” [28]

4 IBM^®^ SPSS^®^ Statistics V26.0.0.0.

5 the Raw data discussed in this paper was partially presented as a poster at the 30th international symposium on ballistics 2017 in long beach, CA [29].

6 In the UK since the introduction of the UK body armour standards using RP1, no UK police officer has lost their life because of BABT or failure of a body armour.
